# Obesity and older age as protective factors for vaginal cuff dehiscence following total hysterectomy

**DOI:** 10.1007/s10397-015-0882-8

**Published:** 2015-01-30

**Authors:** Nicole M. Donnellan, Suketu Mansuria, Nancy Aguwa, Deirdre Lum, Leslie Meyn, Ted Lee

**Affiliations:** 1 Obstetrics, Gynecology and Reproductive Sciences, University of Pittsburgh, Magee-Womens Hospital of UPMC, 300 Halket Street, Pittsburgh, PA 15213 USA; 2 School of Medicine, University of Texas Health Science Center at San Antonio, 7703 Floyd Curl Drive, San Antonio, TX 78229 USA; 3 Obstetrics and Gynecology, Stanford University, 900 Blake Wilbur Drive, Palo Alto, CA 94304 USA; 4 Obstetrics, Gynecology and Reproductive Sciences, Magee-Womens Research Institute, 204 Craft Ave, Pittsburgh, PA 15213 USA

**Keywords:** Dehiscence, Hysterectomy, Laparoscopy, Robotic surgery, Obesity

## Abstract

Studies have shown an increased risk of vaginal cuff dehiscence following total laparoscopic hysterectomy (TLH). Patient variables associated with dehiscence have not been well described. This study aims to identify factors associated with dehiscence following varying routes of total hysterectomy. This is a retrospective, matched, case-control study of women who underwent a total hysterectomy at a large, urban, university-based teaching hospital from January 2000 to December 2011. Women who underwent a total hysterectomy and had a dehiscence (*n* = 31) were matched by surgical mode to the next five total hysterectomies (*n* = 155). Summary statistics and conditional logistic regression were performed to compare cases to controls. Obese women (BMI ≥ 30) were 70 % less likely than normal weight women (BMI < 25) to experience a dehiscence (*p* = 0.02). When stratified by hysterectomy route, obese women were 86 % less likely to have a dehiscence following robotic-assisted total hysterectomy (RAH) and TLH than normal weight women (*p* = 0.04). Further, increasing age was protective of dehiscence in this subgroup of women (*p* = 0.02). Older age and obesity were associated with a decreased risk of dehiscence following RAH and TLH but not following other routes. Increased risk of dehiscence following TLH observed in previous studies may be partially due to patient characteristics.

## Background

As the number of laparoscopic hysterectomies performed each year increases [1, 2], complications associated with this procedure are becoming more evident. One such complication, vaginal cuff dehiscence, has been studied extensively, and numerous reports have described rates of dehiscence to be higher in patients undergoing total laparoscopic hysterectomy (TLH) and robotic-assisted total hysterectomy (RAH) compared to the abdominal or vaginal route [3–9]. This complication is concerning for many gynecologists given the potential morbidity associated with dehiscence, such as peritonitis, sepsis, bowel evisceration, and need for further surgical intervention.

Surgical factors unique to laparoscopic hysterectomy, compared to the abdominal or vaginal route, have been postulated to contribute to the increased risk of dehiscence. Theories include increased tissue damage to the vagina from the use of thermal energy (i.e., monopolar and/or bipolar energy) for colpotomy creation and hemostasis as well as inferior cuff closure due to techniques of laparoscopic suturing [4, 10]. While attempts to investigate the relationship of these factors with dehiscence have been reported, results in the literature remain controversial [11–13] and confusion surrounding the etiology of vaginal cuff dehiscence persists.

Despite overwhelming interest and investigation into the topic of vaginal cuff dehiscence, one variable that has not been well described in previous studies is the patient. Studies have shown that demographic factors such as obesity, smoking, and advanced age are associated with an increased risk of wound breakdown and wound infection; however, these studies focused on open abdominal incisions [14, 15]. The goal of our study was to evaluate if patient-related variables may play a role in the healing of the vaginal cuff. Through performing a retrospective, case-control study, we aimed to discern if, in addition to surgical factors, specific demographic and clinical variables predispose patients to vaginal cuff dehiscence following varying modes of total hysterectomy.

## Methods

This study was a retrospective, matched, case-control study that examined demographic, clinical, and surgical characteristics of women who experienced a vaginal cuff dehiscence following total hysterectomy compared to women who did not experience this complication. Institutional review board approval was obtained from the University of Pittsburgh prior to initiation of the study. Pertinent *Physicians’ Current Procedural Terminology Coding System*, 4th edition (CPT-4) procedure codes and *International Classification of Diseases*, 9th revision diagnostic codes were used to identify all women who had a repair of a vaginal cuff dehiscence at Magee-Womens Hospital, which is a large, urban, university-based teaching hospital, between January 2000 and December 2011. Surgeons included general gynecologists, gynecologic oncologists, urogynecologists, and gynecologists trained in minimally invasive gynecologic surgery. Patients were excluded if their primary surgery (hysterectomy) was not performed at our institution.

Cases of vaginal cuff dehiscence were matched 1:5 temporally and by route of hysterectomy. Thus, for each case of dehiscence, diagnostic codes were used to identify the next five uncomplicated hysterectomies performed at Magee by the same route as the case. We controlled for route of hysterectomy as previous studies have shown an increased risk of vaginal cuff dehiscence following TLH compared to a vaginal or abdominal approach [3–9]. Five controls per case were obtained to maximize study size while appreciating that minimal statistical power is gained by increasing this ratio beyond 1:4 or 1:5 [16].

Vaginal cuff dehiscence was defined as partial or complete separation of the vaginal cuff with or without evisceration. TLHs and robotic-assisted laparoscopic hysterectomies (RAHs) in our study were defined as hysterectomies performed entirely through a laparoscopic or robotic approach, including colpotomy and cuff closure [17]. Hysterectomies performed laparoscopically but with a vaginal colpotomy and/or cuff closure were categorized as laparoscopic-assisted vaginal hysterectomy (LAVH). As a separate diagnostic code did not exist to distinguish TLH and RAH from LAVH for a majority of the study period, appropriate classification of hysterectomy type was confirmed by operative note review.

A chart review of paper and electronic medical records was performed to obtain demographic, clinical, and surgical characteristics of all cases and controls. Demographic information obtained included age, body mass index (BMI), and race. Clinical characteristics included parity, tobacco use, menopausal status, and diagnosis of hypertension or diabetes. Preoperative diagnoses, postoperative pathology, and intraoperative details, such as estimated blood loss (EBL), case length, uterine specimen weight, and techniques of colpotomy creation and cuff closure, were also recorded.

Statistical analyses were performed using Stata statistical software version 11.2 (StataCorp LP, College Station, Texas, USA), and statistical tests were evaluated at the two-sided 0.05 significance level. The association between vaginal cuff dehiscence and demographic, clinical, and surgical characteristics was assessed using conditional logistic regression to estimate odds ratios (OR) and 95 % confidence intervals (CI) both in univariable and multivariable models. A subanalysis was performed stratifying based upon route of hysterectomy.

## Findings

From January 2000 through December 2011, there were 31 cases of vaginal cuff dehiscence that presented to Magee-Womens Hospital following a total hysterectomy initially performed at our institution. These cases were matched, as previously described, to 155 uncomplicated controls. Among the dehiscence cases, 13 (41.8 %) presented after TLH, 12 (38.7 %) after total abdominal hysterectomy (TAH), 2 (6.5 %) after total vaginal hysterectomy (TVH), 2 (6.5 %) after LAVH, and 2 (6.5 %) after RAH.

Vaginal cuff dehiscence cases had a mean age of 45.0 years (SD 13.4), mean BMI of 27.0 kg/m^2^ (SD 6.7), and mean parity of 2.1 (SD 1.4). Controls had a mean age of 47.9 years (SD 12.5), mean BMI of 30.1 kg/m^2^ (SD 7.4), and mean parity of 1.7 (SD 1.3). Seven cases (22.6 %) and 26 controls (16.8 %) had a preoperative diagnosis of cancer. Surgical characteristics of cases included a mean EBL of 258.4 cc (SD 426.6), mean operative length of 141 min (SD 51), and mean uterine weight of 182.8 g (SD 161.6). Controls had a mean EBL of 217 cc (SD 234.4), mean operative length of 141 min (SD 61), and mean uterine weight of 186.1 g (SD 172.5). Further demographic, clinical, and surgical characteristics are summarized in Table 1.

**Table 1:** Association of demographic and clinical factors with vaginal cuff dehiscence following total hysterectomy

Factor	Cases (*n* = 31)	Controls (*n* = 155)	Unadjusted OR (95 % CI)	*p* value^a^
Age (years)	45.0 ± 13.4	47.9 ± 12.5	0.98 (0.95–1.01)	0.23
Body mass index (kg/m^2^)				
<25	14 (45.2)	45 (29.2)	1.0 (referent)	
≥25 to <30	10 (32.3)	40 (26.0)	0.81 (0.33–1.99)	0.64
≥30	7 (22.6)	69 (44.8)	0.29 (0.10–0.83)	0.02*
Race				
African-American	6 (19.4)	20 (12.9)	1.69 (0.59–4.91)	0.33
Parity				
≥ 1	26 (83.9)	119 (78.3)	1.47 (0.50–4.30)	0.48
Tobacco	11 (35.5)	38 (24.7)	1.92 (0.76–4.88)	0.17
Menopause	9 (30.0)	50 (32.5)	0.83 (0.34–2.01)	0.68
Hypertension	8 (25.8)	39 (25.16)	1.03 (0.43–2.49)	0.94
Diabetes	3 (9.7)	10 (6.5)	1.58 (0.40–6.27)	0.52
Indication for surgery				
Pelvic pain	12 (38.7)	61 (39.4)	0.97 (0.43–2.20)	0.95
Abnormal uterine bleeding	14 (45.2)	70 (45.2)	1.00 (0.45–2.24)	>0.99
Fibroids	7 (22.6)	36 (23.2)	0.96 (0.34–2.65)	0.93
Endometrial cancer	4 (12.9)	24 (15.5)	0.81 (0.26–2.52)	0.71
Endometriosis	3 (9.7)	12 (7.7)	1.30 (0.32–5.13)	0.71
Prolapse	2 (6.5)	8 (5.2)	1.34 (0.22–8.05)	0.75
Malignant pathology	7 (22.6)	32 (20.8)	1.15 (0.42–3.16)	0.79
Monopolar colpotomy	16 (51.6)	75 (66.4)	1.33 (0.33–5.30)	0.69
Suture for cuff closure				
Polysorb	18 (78.3)	84 (65.6)	2.53 (0.58–11.09)	0.22
PDS	2 (8.7)	11 (8.6)	0.86 (0.13–5.53)	0.88
Barbed	1 (4.4)	8 (6.3)	0.47 (0.04–5.68)	0.56
Estimated blood loss (cc)				
≥200	14 (45.2)	64 (41.3)	1.28 (0.49–3.39)	0.62
Length of case (minutes)				
≥120	11 (35.5)	58 (37.4)	0.91 (0.40–2.10)	0.91
Uterine weight (g)				
≥200	9 (30.0)	37 (24.2)	1.35 (0.55–3.27)	0.51

Data presented as mean ± SD for continuous variables or *n* (%) for dichotomous variables

*OR* odds ratio, *CI* confidence interval

*p < 0.05, statistically significant

^a^ *p* value from unadjusted conditional logistic regression model

When comparing cases to controls, obese women (BMI ≥ 30) were 70 % less likely than normal weight women (BMI < 25) to experience a dehiscence (*p* = 0.02). All other demographic, clinical, and surgical factors examined were not associated with vaginal cuff dehiscence (*p* > 0.05, Table 1).

A subanalysis was performed after stratifying cases and controls based upon route of surgery (TLH and RALH versus VH, LAVH, and TAH; Table 2). Following stratification, overweight (BMI 25–29) women were 80 % less likely than normal weight women (BMI < 25) to have a dehiscence following RAHs and TLHs (*p* = 0.04; Table 2). Obese women were 86 % less likely to have a dehiscence following RAHs and TLHs than normal weight women (*p* = 0.04; Table 2). Further, increasing age was protective of a dehiscence event in this subgroup of women who underwent RAHs or TLHs (*p* = 0.02; Table 2). Age and BMI were not significantly associated with dehiscence in the other routes (*p* > 0.05, Table 2). Race was the only factor associated with dehiscence following LAVH, TVH, and TAH. In this hysterectomy subgroup, black women had a four-fold increased risk of dehiscence compared to other races (*p* = 0.03; Table 2).

**Table 2:** Association of demographic factors with vaginal cuff dehiscence following total hysterectomy stratified by mode of hysterectomy

Factor	Cases	Controls	Unadjusted OR (95 % CI)	*p* value^a^	Adjusted^b^ OR (95 % CI)	*p* value^a^
Total laparoscopic and robotic hysterectomy	*N* = 15	*N* = 75				
Age (years)	38.2 ± 7.8	46.1 ± 12.4	0.92 (0.86–0.99)	0.02*	0.90 (0.82–0.98)	0.02*
Body mass index (kg/m^2^)						
<25	10 (66.7)	23 (30.7)	1.0 (referent)	–	1.0 (referent)	–
≥25 to <30	3 (20.0)	23 (30.7)	0.31 (0.08–1.23)	0.10	0.19 (0.04–0.90)	0.04*
≥30	2 (13.3)	29 (38.7)	0.16 (0.03–0.80)	0.03*	0.14 (0.02–0.92)	0.04*
Other routes of total hysterectomy	*N* = 16	*N* = 80				
Age (years)	51.4 ± 14.5	49.7 ± 12.5	1.01 (0.97–1.05)	0.62		
Body mass index (kg/m^2^)						
<25	4 (25.0)	22 (27.9)	1.00 (referent)	–		
≥25 to <30	7 (43.8)	17 (21.5)	2.43 (0.57–10.41)	0.23		
≥30	5 (31.3)	40 (50.6)	0.62 (0.14–2.83)	0.54		
Race						
African-American	6 (37.5)	11 (13.8)	4.34 (1.16–16.27)	0.03*		

Data presented as mean ± SD for continuous variables or *n* (%) for dichotomous variables

*OR* odds ratio, *CI* confidence interval

*p < 0.05, statistically significant

^a^ *p* value from conditional logistic regression model

^b^Adjusted for age and body mass index

## Conclusion

To our knowledge, this is the first study to demonstrate that patient characteristics of age, BMI, and race are significantly associated with vaginal cuff dehiscence following total hysterectomy. Recent literature has voiced concern regarding the increased risk of vaginal cuff dehiscence following TLH, with a reported incidence as high as 4.6 % [4]. A major shortcoming of these studies is the lack of information detailing the patients undergoing the various types of total hysterectomy. Thus, we designed a case-control study matched on mode of hysterectomy and found that obesity is associated with a significant decrease in dehiscence. After stratifying by hysterectomy type, we found that in the subpopulation of women undergoing a TLH or RAH, this association remains significant. In addition, being overweight and increasing age were also independently associated with a decrease in dehiscence following the laparoscopic and robotic modes.

Our findings suggest that the increased risk of vaginal cuff dehiscence following TLH and RAH may not be entirely explained by the surgical characteristics inherent to these procedures, as has been previously theorized in the literature. Instead, we have shown that patient characteristics may account for, at least partially, the increased rate of dehiscence following a laparoscopic approach. We hypothesize that obesity is protective against dehiscence following TLH and RALH secondary to both structural and physiologic properties. Act of first coitus following total hysterectomy has been a commonly identified inciting event for vaginal cuff dehiscence [4, 5, 8, 10, 11]. Physical forces at the apex of the vaginal cuff during intercourse may be decreased in more obese women, thus providing a protective effect. Similarly, positioning during intercourse may be different in obese patients as compared to normal weight individuals (i.e., as in pregnant patients), which may also confer decreased physical force along the healing vaginal cuff. Older women may experience decreased postoperative activity, including intercourse, which may explain the protective effect demonstrated in this study. However, such proposed theories need to be further examined through studies involving detailed patient questionnaires to better elucidate specific postoperative activity patterns.

Past studies demonstrating an increased risk of dehiscence following TLH have suggested that the technique of monopolar electrosurgery may predispose patients to dehiscence. While we did not demonstrate an association between dehiscence and technique of colpotomy, obesity may confer a protective effect through the physiologic property of increasing impedance in the monopolar circuit. With increased impedance owing to increased adipose tissue in the circuit, less total energy is delivered to a given area, which may lead to less tissue destruction at time of colpotomy. Further studies detailing the thermal effect of electrosurgery as it relates to tissue type are warranted.

Our findings of obesity and increasing age being associated with cuff dehiscence following TLH or RALH could have major implications on clinical practice patterns in minimally invasive gynecologic surgery. If surgeons at our institution had performed laparoscopic supracervical hysterectomies instead of TLHs on all patients with a BMI <25 kg/m^2^ over the past 10 years, the dehiscence rate would be estimated to be 0.4 %, a greater than three-fold risk reduction from our most recent reported incidence of 1.4 % [8]. However, such a radical practice change does not take into account other clinical reasons and indications, in addition to economic factors, for performing a total versus a supracervical hysterectomy. Supracervical hysterectomy is not without associated morbidities and costs, including cervical stump bleeding and the need for continued cervical cancer screening [18–21]. Further, reoperation for trachelectomy has been reported to be as high as 22 % [21]. Such a proposed practice change may simply reduce the incidence of one specific complication while giving rise to others and may not reduce overall patient morbidity.

Analysis of our stratified data also revealed that African-American race was associated with a greater than four-fold increased risk of dehiscence following vaginal and abdominal procedures. One must be cautioned prior to making any conclusions about this result, as less than 15 % of the entire study population identified themselves as African-American. This already low prevalence can lead to erroneous conclusions when attempting to further stratify and analyze data. Race may also be a marker for other demographic or behavioral risk factors. Analysis of a more heterogeneous population in regard to race is merited prior to forming definitive conclusions regarding the impact of race on vaginal cuff dehiscence.

A major limitation to this study is the difficulty in designing a feasible yet valid study focusing on an event with an extremely low incidence. At our institution, greater than 1000 total hysterectomies are performed each year, yet only 31 vaginal cuff dehiscences in an 11-year period were identified, producing a dehiscence rate of less than 0.3 % [5]. While a case-control design assists in studying rare events or conditions, such study designs have inherent biases including sampling and case ascertainment [22]. The rarity of a dehiscence event also makes stratification and statistical analysis by single mode of hysterectomy impossible with our current data set. Thus, while we have grouped similar modes of hysterectomy together and performed a subanalysis (TLH and RAH versus TAH, TVH, and LAVH), there may be intrinsic procedure-specific risks related to dehiscence on which we are unable to comment owing to the extremely low event rate. Another limitation to this chart review is the difficulty in providing accurate correction for rare, patient characteristics, such as prior pelvic radiation, which may confound dehiscence susceptibility. Further, a single laparoscopic radical hysterectomy was grouped with the TLHs as there were limitations in obtaining laparoscopic radical hysterectomy controls. Such a procedure with aggressive pelvic dissection may also have inherent surgical characteristic which could predispose a patient to dehiscence. In addition, although our study is from a high-volume, tertiary care facility, another limitation is the narrow geographic range and its generalizability to other patient populations (i.e., external validity). Overcoming such limitations underscores the importance of future multicenter collaborations in order to increase both absolute dehiscence event numbers as well as patient heterogeneity.

In addition to larger, multicentered collaborations, future studies should also focus on examining the proposed theories we have highlighted to explain the protective effect of obesity and older age on vaginal cuff dehiscence. Such studies should include patient-based investigations focusing on detailed postoperative activities and habits, as well as basic-science studies examining tissue strength and integrity. Until such studies are conducted, however, we present novel data that can assist the gynecologic surgeon to better preoperatively identify patients at increased risk of dehiscence and counsel them accordingly, with the ultimate goal of optimizing surgical outcomes.
